# Automatic and standardized quality assurance of digital mammography and tomosynthesis with deep convolutional neural networks

**DOI:** 10.1186/s13244-023-01396-8

**Published:** 2023-05-18

**Authors:** Patryk Hejduk, Raphael Sexauer, Carlotta Ruppert, Karol Borkowski, Jan Unkelbach, Noemi Schmidt

**Affiliations:** 1 Institute of Diagnostic and Interventional Radiology, University Hospital Zurich, Rämistr. 100, 8091 Zurich, Switzerland; 2 Breast Imaging, Radiology and Nuclear Medicine, University Hospital Basel, Basel, Switzerland; 3 Department of Radiation Oncology, University Hospital Zurich, Zurich, Switzerland

**Keywords:** Mammography, Tomosynthesis, Quality control, Machine learning, Artificial intelligence

## Abstract

**Objectives:**

The aim of this study was to develop and validate a commercially available AI platform for the automatic determination of image quality in mammography and tomosynthesis considering a standardized set of features.

**Materials and methods:**

In this retrospective study, 11,733 mammograms and synthetic 2D reconstructions from tomosynthesis of 4200 patients from two institutions were analyzed by assessing the presence of seven features which impact image quality in regard to breast positioning. Deep learning was applied to train five dCNN models on features detecting the presence of anatomical landmarks and three dCNN models for localization features. The validity of models was assessed by the calculation of the mean squared error in a test dataset and was compared to the reading by experienced radiologists.

**Results:**

Accuracies of the dCNN models ranged between 93.0% for the nipple visualization and 98.5% for the depiction of the pectoralis muscle in the CC view. Calculations based on regression models allow for precise measurements of distances and angles of breast positioning on mammograms and synthetic 2D reconstructions from tomosynthesis. All models showed almost perfect agreement compared to human reading with Cohen’s kappa scores above 0.9.

**Conclusions:**

An AI-based quality assessment system using a dCNN allows for precise, consistent and observer-independent rating of digital mammography and synthetic 2D reconstructions from tomosynthesis. Automation and standardization of quality assessment enable real-time feedback to technicians and radiologists that shall reduce a number of inadequate examinations according to PGMI (Perfect, Good, Moderate, Inadequate) criteria, reduce a number of recalls and provide a dependable training platform for inexperienced technicians.

## Key points

1. Deep convolutional neural network (dCNN) models have been trained for classification of mammography imaging quality features.
2. AI can reliably classify diagnostic image quality of mammography and tomosynthesis.
3. Quality control of mammography and tomosynthesis can be automated.

## Introduction

Every year, 2.3 million women are diagnosed with breast cancer [1] representing 25% of all cancer cases in women [2]. Due to a relative lack of symptoms in the initial phases, early detection of breast cancer is important for mortality reduction [3]. Mammography breast cancer screening is the most common measure for mortality reduction, proved to lower death rates by 24–48% in controlled trials [4–6]. Although providing tangible benefits, screening is not a perfect solution. Risk of over-diagnosis, lack of automated tools for comprehensive evaluation of diagnostic quality, moderate accuracy in patients with dense breasts as well as high healthcare costs of breast cancer screening programmes are serious threats to the legitimacy of screening programmes [7].

For successful radiological evaluation of mammograms and tomosynthesis images, sufficient diagnostic information depicting as much breast parenchyma as possible has to be present on the two projection images. In screening programmes, quality assurance (QA) of physical and technical aspects is implemented, providing repeatable and standardized evaluations of image quality that are comparable between diagnostic units. Over the years, several QA protocols have been developed, although there is incomplete harmonization between them [8, 9]. The diagnostic image quality of an examination has a significant impact on cancer detectability [10]: inadequate positioning, image artefacts or insufficient breast compression may reduce the sensitivity for the detection of breast cancer from 84.0 to 66.3% [11]. In Europe and the USA, the most common diagnostic image quality control of mammographies are the Perfect–Good–Moderate–Inadequate (PGMI) criteria created by the United Kingdom Mammography Trainers group with the support of the Society and College of Radiographers [12], which recommend a qualitative evaluation of the diagnostic image quality to be performed by the radiologists or by a specially trained radiographer. Typically, in quality-controlled screening programmes, 70% perfect or good mammographies are requested and less than 3% inadequate mammographies are acceptable [13], in some screening programmes even stricter rules are applied with more than 75% perfect or good mammographies [14].

Even though the PGMI criteria are well defined, subjectivity of the human assessment may result in low reliability of the rating because of inter-reader variability, and it has been reported that the inter-reader agreement ranges only between slight (*k* = 0.02) and fair (*k* = 0.40) [15]. One study suggests that even 49.7% of mammograms do not satisfy the requested quality criteria of screening programmes resulting in additional and unnecessary risks for patients [15]. On top of that, each of those tasks is time-consuming, tedious and prone to errors from external factors for human readers [16].

Standardization of methods and automation of processes may have a strong impact on the image quality of mammography examinations. Thereby, standardization and automation potentially result in an increase of capacities of organizations, improved cancer detection rates, reduced diagnostic errors and a substantial decrease in healthcare costs [17]. QA automation with artificial intelligence constitutes a viable solution to those problems, minimizing errors in quality ratings and increasing general productivity in the mammography unit [18], although there is very limited commercial availability of products that are able to perform analysis of single-quality features providing explainable feedback to the end-user.

Artificial intelligence has been proven to accurately classify the breast density in mammography according to the ACR BI-RADS standard [19, 20]. Moreover, dCNNs have been used to mimic human decision-making process in the detection and classification of lesions in ultrasound imaging [21] as well as recognizing subtle features in mammographies like microcalcifications [22]. Although institutions performing screening programmes are adopting AI-based solutions, automated image quality control is not routinely available.

In this study, we trained dCNN models to determine the presence and the location of quality features in mammographies and tomosynthesis examinations, and we compared the accuracies of the dedicated dCNN models to human expert reading.

## Materials and methods

### Patient data

This retrospective study has been approved by the local ethics committee (“Kantonale Ethikkommission Zurich”; Approval Number: 2016–00,064 and Ethikkommission Nordwest-und Zentralschweiz, Project ID: 2021–01,472).

The included images were acquired between 2012 and 2020, the whole time span included in our ethics committee approval. The images originated from five different devices (manufactured by Siemens, Hologic, GE, IMS Giotto and Fujifilm) located in two institutions. All devices used in the study were running according to quality control tolerances and standards. Only female patients were included between 20 and 97 years old (median 57 years old).

We excluded all images with the presence of foreign objects like silicone implants, pacemakers and other foreign bodies like surgical clips, 662 images in total (478 DM, 184 DT). Postoperative patients were excluded as well.

For model training and validation, 90% of the included images were used (70% for training, 20% for validation), in detail 11,733 digital mammography (DM) images and synthetic 2D reconstructions of digital breast tomosynthesis (DBT) of 4200 female patients (CC orientation: 3565 DM, 2873 DT; MLO orientation: 2,710 DM, 2585 DT). For testing of classification models, 10% of the images were used; a separate dataset from training and validation datasets, a dataset consisting of 1,944 images, was used (CC orientation: 369 DM, 602 DT; MLO orientation: 371 DM, 602 DT). The testing of the regression models has been performed on 388 images (10% of images with position labels; CC orientation: 68 DM, 110 DT; MLO orientation: 82 DM, 128 DT).

### Data preparation

Images for training and validation of dCNN models have been retrieved from the institutions’ PACS systems. Due to differences in image properties of the various manufacturers, each image was pre-processed. To avoid distortions, images from left orientation were flipped horizontally to keep breast projection on one side of an image for further post-processing. Differences in image sizes and aspect ratios were addressed by adding a stripe of black background colour to the right side of the image to create an image with square dimensions. Differences in image brightness and contrast between devices were controlled by applying windowing based on the manufacturer’s look-up tables. All images have been saved in lossless image format to avoid image quality degradation due to compression or reduction of bit depth. Images for nipple classification were cropped around the position of the nipple, which was marked by radiologists containing 10% of the original image’s width and height.

### Image selection and data preparation

**Fig. 1:**
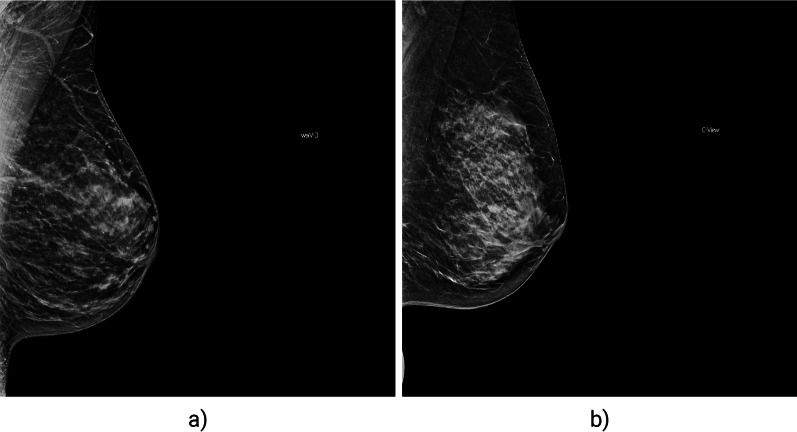
Example of IMF feature classification; **a** feature present, **b** feature missing

**Fig. 2:**
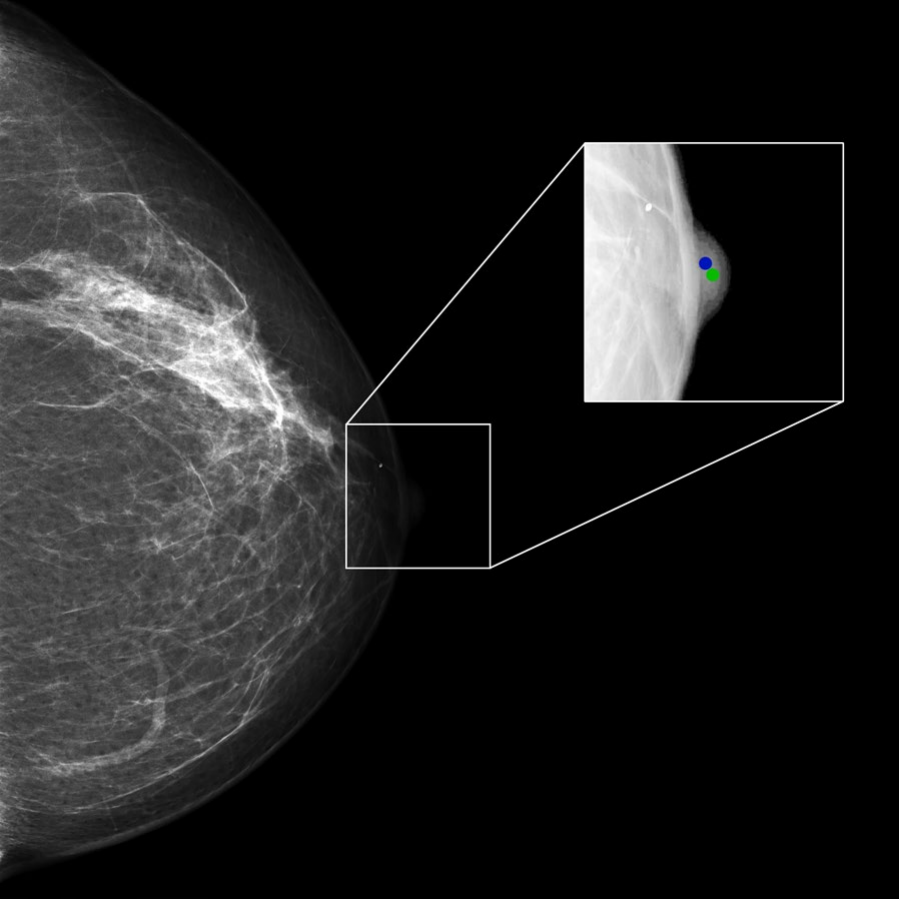
Example of regression; green dot shows nipple position marked by a radiologist; blue dot shows the position predicted by dCNN

For determination of the image quality regarding the breast positioning, two methods were used: classification to determine feature presence and regression for precise localization of a feature on the image. In the classification task, four radiologists, with at least 3–8 years of experience in mammography imaging, independently labelled images by determining whether the respective feature is visible on an image or missing. An example of a classification of an IMF (inframammary fold) feature is presented in Fig. 1. For the regression tasks, the radiologists marked the location of a feature on the image. An example of the labelling of a position by a radiologist and the corresponding prediction of the AI model are presented in Fig. 2. All tasks related to image labelling were performed by two radiologists with at least 3 and 8 years of experience in breast imaging after the board certification, respectively.

### Model selection

Eight dCNN models were trained to assess key image quality impacting the overall diagnostic image quality assessment: five classification models for detecting the presence of anatomical landmarks or features and three regression models for feature localization.

#### Classification models

1. *Parenchyma depiction in CC and MLO orientation* determination if the whole breast parenchyma is present on an image in CC view;
2. *Parenchyma depiction in MLO orientation* determination if the whole breast parenchyma is present on an image in MLO view;
3. *Inframammary fold (IMF) in MLO orientation*: presence of inframammary fold on the image. If fold is missing, there is also no certainty that the chest is adequately shown and corresponding tissue cannot be assessed;
4. *Pectoralis muscle in CC orientation* presence of the pectoralis muscle on a CC view to ensure that tissue near the chest wall is correctly recorded;
5. *Nipple depiction in both CC and MLO views* determination if the nipple is in profile of images of both projections and if the nipple is centered on the CC view.

#### Regression models

(6) Position of the nipple in both CC and MLO views;
(7) *Pectoralis cranial in MLO view* position of the pectoralis muscle at the cranial edge of the image (cranial point);
(8) *Pectoralis caudal in MLO view* position of the pectoralis muscle at the thoracic wall at the edge of the image (caudal point).

**Fig. 3:**
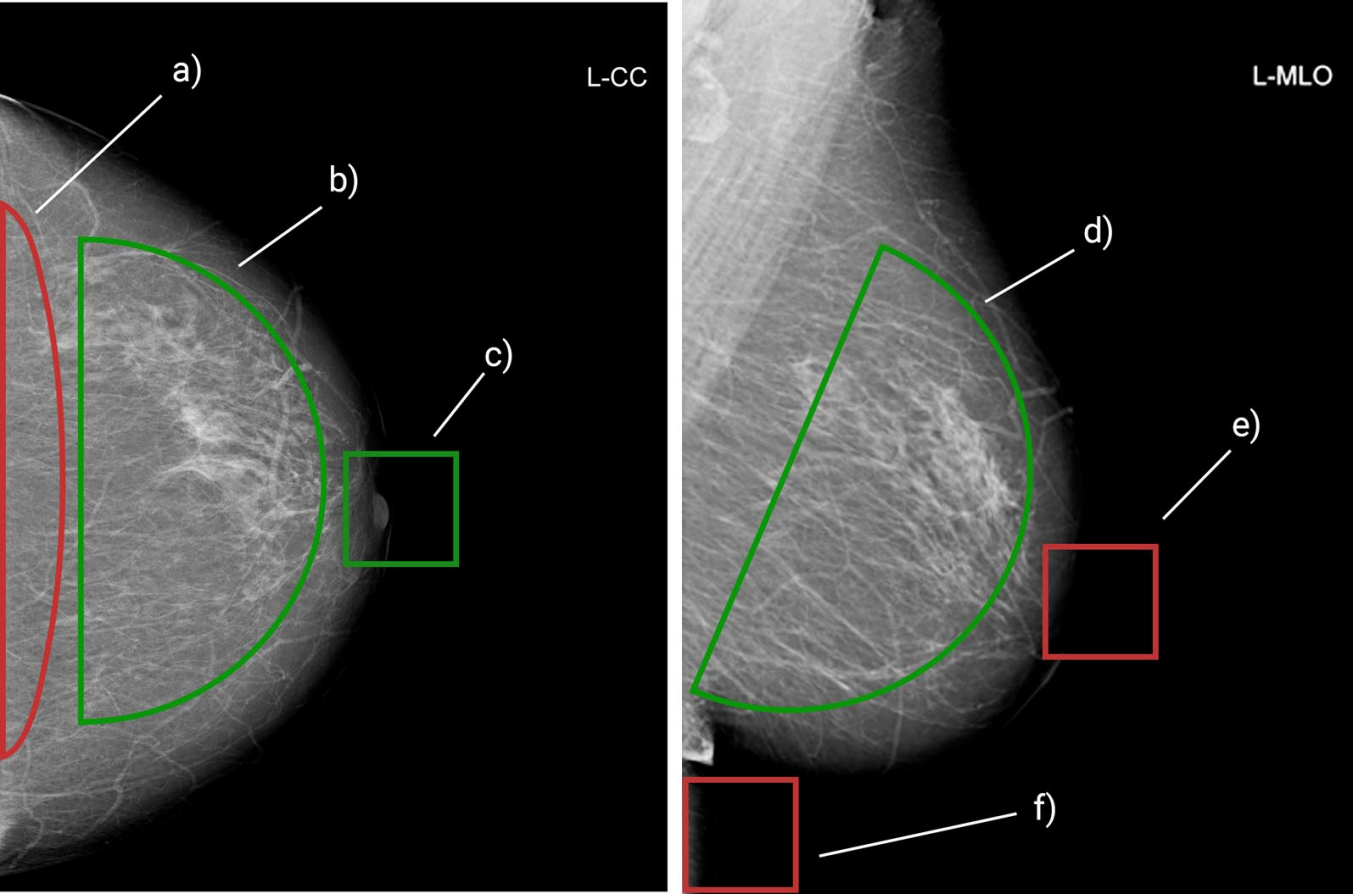
Example of full breast qualitative features classification; **a** no pectoralis muscle depiction; **b** parenchyma on CC image is sufficiently depicted; **c** nipple on CC image is in profile; **d** parenchyma on MLO image is sufficiently depicted; **e** nipple on MLO image is not in profile; **f** Inframammary fold is not depicted sufficiently

**Fig. 4:**
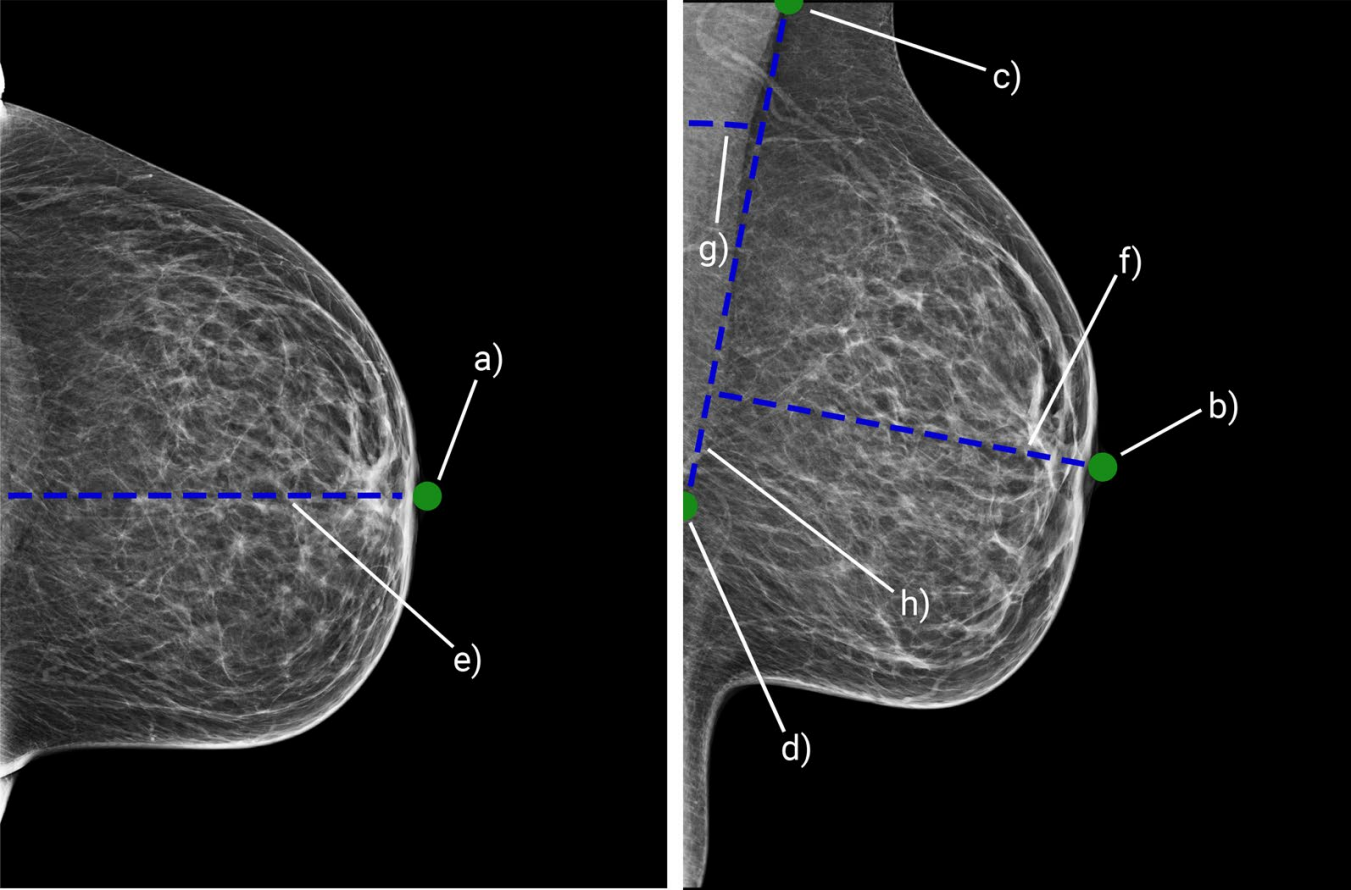
Example of full breast quantitative features detection; detection of a nipple position on CC image (**a**) and MLO image (**b**); detection of cranial (**c**) and caudal (**c**) positions of a pectoralis muscle on an image; calculations of pectoralis–nipple lengths on CC (**e**) and MLO image (**f**); calculations of pectoralis angle (**g**); calculation of pectoralis–nipple level

An example of a qualitative evaluation of an examination is presented in Fig. 3. Anatomical landmarks found by regression models allow for the determination of the angle, in which the pectoral muscle is depicted and the calculation of the posterior nipple line in CC compared to MLO. The pectoralis angle was assessed with the use of two object detection models: detection cranial and thoracic wall edges position of the pectoral muscle compared to the evaluation performed by both experienced radiologists. The posterior nipple line length was calculated using results from the three object detection models: nipple detection in both projections and cranial and caudal points in MLO projection to assure that parts of the parenchyma near the pectoral muscle may are adequately represented. Examples of detected points and calculations are presented in Fig. 4.

Images have been randomly split into training and validation datasets in proportions of 3:1. The exact numbers of images which were used for training of each model are presented in Table 1. All images were rescaled from the original resolution to squares 224 × 224 pixels; to improve the model generalization, training and validation datasets were randomly augmented using the TensorFlow (Google LLC) data augmentation tool for rotation, horizontal, vertical shifts and zooming. The class weighting method was used to counteract class imbalances.

**Table 1:** Results of models training and validation

Type	MODEL	Projection	Training images	Validation images	Training accuracy /training RSME [mm]	Validation accuracy /validation RSME [mm]
Classification	Parenchyma	CC	4750	1357	89.4%	87.2%
	Parenchyma	MLO	3861	1103	88.5%	86.9%
	IMF	MLO	2826	807	84.6%	84.9%
	Pectoralis	CC	1255	359	88.1%	92.7%
	Nipple	CC + MLO	1155	330	87.3%	86.9%
Regression	Nipple	CC + MLO	2720	777	8.3	8.4
	Pectoralis cranial	MLO	1467	419	8.2	12.9
	Pectoralis caudal	MLO	1467	419	8.3	8.4
	Pectoralis angle	MLO	1467	419	n/a	n/a
	Posterior-Nipple line	MLO	1467	419	10.5	11.3

Results for Pectoralis Angle and Posterior-Nipple Line have been calculated based on Nipple and Pectoralis positions

### dCNN architecture

Classification models have been trained with a network containing 13 convolutional layers with 3 × 3 kernel size with five max-pooling layers in between for dimension reduction and two dense layers with rectified linear unit activation function. Batch normalization and 50% dropout have been used for overfitting reduction. For final weights, softmax layer was used. Stochastic gradient descent was used for optimization and cross-entropy as loss function.

The regression models used similar architecture, but the loss function was replaced with a mean squared error (MSE), and Sigmoid was used as an activation function. Input for the regression model was a tuple containing an image and coordinates of a marked point presenting the position of a desired feature.

Training has been done on batches of 32 images with a learning rate of 1.0 × 10^−5^, and dCNN models were trained for 160 epochs, with a new model being saved after each epoch. The dCNN model with the highest validation accuracy or lowest validation RMSE has been chosen for testing. dCNN models were trained on a machine equipped with an Nvidia 1080 GTX GPU running a TensorFlow 2.0 software library on Ubuntu 16.04 OS.

### Image analysis

All image analyses were done with a commercial platform (“b-box”, v 1.1, b-rayZ AG), a framework for automatic analyses of mammography images and synthetic 2D reconstructions from tomosynthesis. Test images were uploaded to the DICOM server of the b-box and automatically evaluated with the above-described dCNN models. The results of dCNN models’ predictions have been compared with classifications and regressions performed by radiologists as ground truth. The results evaluation for the classification tasks has been performed using the accuracy metric comparing class outcome of the model with the class outcome labelled by the radiologist. Regression models predicted coordinates of the feature position in comparison with the feature position marked by the radiologist, and the RMSE metric was used for error calculation. Images were analysed on a diagnostic monitor (EIZO RX350, resolution: 1536 × 2048, EIZO).

### Statistical analysis

Confidence intervals (CIs) for prediction accuracies were calculated using the Monte Carlo method based on the distribution of prediction probabilities or errors for the test datasets. The statistical analysis was performed using the Scikit-learn 0.22.1 package for the Python programming language. Predictions of both accuracies and RMSE were evaluated with a confidence interval (CI) = 0.95.

## Results

### Training, validation and test results

Training of classification dCNN models resulted in accuracies between 84.6 and 89.4% for training images and 84.9–92.7% on the validation datasets. Regression models showed RMSEs ranging from 8.2 to 10.5 mm for training data and from 8.4 to 12.9 mm for the validation data. The detailed results of the training and validation accuracies and errors are presented in Table 1. The evaluation of results on test datasets is presented in Table 2.

**Table 2:** Results of model accuracies and errors on test datasets

Type	MODEL	Projection	Test images	Test accuracy/test RSME [mm]	Sensitivity TPR	Specificity TNR	Precision PPV
Classification	Parenchyma	CC	1944	97.1% [95% CI 96.2–97.7%]	99.8% [95% CI 99.3–99.9%]	95.4% [95% CI 93.7–96.3%]	93.4% [95% CI 91.6–94.8%]
	Parenchyma	MLO		87.3% [95% CI 86.3–88.2%]	89.0% [95% CI 87.4–90.2%]	87.6% [95% CI 85.0–89.2%]	93.6% [95% CI 91.7–94.8%]
	IMF	MLO	973	96.3% [95% CI 94.9–97.4%]	99.3% [95% CI 98.2–99.7%]	93.1% [95% CI 90.4– 95.2%]	93.6% [95% CI 91.4–95.3%]
	Pectoralis	CC	971	98.5% [95% CI 98.2–99.5%]	99.5% [95% CI 98.6–99.9%]	96.3% [95% CI 93.7–98.1%]	98.1% [95% CI 96.8–98.9%]
	Nipple	CC + MLO	1944	93.0% [95% CI 91.7–94.0%]	94.0% [95% CI 92.8–95.1%]	69.2% [95% CI 57.9–78.9%]	98.5% [95% CI 98.0–98.9%]
Regression	Nipple	CC + MLO	350	8.4 [95% CI 7.3–9.5]	n/a		
	Pectoralis Cranial	MLO	188	17.9 [95% CI 13.2–21.0]	n/a		
	Pectoralis Caudal	MLO	188	13.2 [95% CI 9.9–16.0]	n/a		
	Pectoralis Angle	MLO	973	99.1% [95% CI 98.2–99.5%]	99.0% [95% CI 98.1–99.6%]	99.0% [95% CI 96.6–99.8%]	99.7% [95% CI 98.9–99.9%]
	Posterior-Nipple Line	MLO	973	99.0% [95% CI 98.1–99.1%]	99.7% [95% CI 98.9–99.9%]	96.9% [95% CI 94.1–98.7%]	98.8% [95% CI 97.8–99.4%]

#### Parenchyma classification

Depiction of parenchyma by AI models was correct in 97.1% [95% CI 96.2–97.7%] of test cases in CC view and in 87.3% [95% CI 86.3–88.2%] of test images in MLO view. In CC view precision was 0.93, recall 0.99 and *F*1-score 0.96. In MLO view precision was 0.94, recall 0.89 and *F*1-score 0.91. Almost perfect agreement with radiologists was achieved with a Cohen’s kappa score of 0.94 [95% CI 0.93–0.96].

#### Inframammary fold classification

Depiction of Inframammary fold in MLO projection was correct on 96.3% [95% CI 94.9–97.4%] of images. Precision of the model was 0.94, recall 0.99 and *F*1-score 0.96. The model showed almost perfect agreement with radiologists with a Cohen’s kappa score of 0.93 [95% CI 0.90–0.95].

#### Pectoralis muscle classification

Depiction of the pectoral muscle in CC projection was correct on 98.5% [95% CI 98.2–99.5%] of images. Precision of the model was 0.98; recall and *F*1-score were 0.99. The model showed almost perfect agreement with radiologists with a Cohen’s kappa score of 0.96 [95% CI 0.94–0.98].

#### Nipple depiction

Depiction and classification of nipple in profile in MLO and nipple in profile and centered in CC images was correct in 93.0% [95% CI 91.7–94.0%] of the evaluated cases. Precision of the model was 0.99, recall 0.94 and *F*1-score 0.96. The model showed moderate agreement with radiologists was achieved with a Cohen’s kappa score of 0.42 [95% CI 0.34–0.50].

### Regression models

The regression model for nipple position detection was able to predict the position with an error of 8.4 mm [95% CI 7.3–9.5 mm].

In MLO view, the cranial point of a pectoralis muscle was detected with an error of 17.9 mm [95% CI 13.2–21.0 mm] and the caudal point with an error of 13.2 mm [95% CI 9.9–16.0 mm].

#### Quality features calculations

The accuracy of the calculation of pectoralis angle was 99.1% [95% CI 98.2–99.5%] and for posterior nipple line 99.0% [95% CI 98.2–99.5%]. Precision, recall and *F*1-score were 0.99 in both cases. The calculations have been compared to the evaluation performed by our experienced radiologists. For the radiologists, accuracy of the calculation was 99.0% and precision, recall and *F*1-score were 0.99. Agreement with radiologists was almost perfect, and Cohen’s kappa score was 0.97 [95% CI 0.95–0.99].

## Discussion

In this study, we were able to show that deep convolutional neural networks can be used to assess quality features on mammograms and synthetic 2D reconstructions from tomosynthesis. Classification models achieved over 85% accuracy for determination of key image quality features in breast imaging. Regression models allowed for precise localization of desired features to indicate possible positioning errors. When comparing results of the test dataset evaluation, all dCNN models were able to achieve almost perfect agreement with the radiologists.

In recent years, AI-based computer-aided detection solutions have attracted the attention of radiologists worldwide. A dCNN is an excellent method for image classification tasks. AI performance in breast cancer detection in mammography is comparable to experienced radiologists [23, 24], despite the struggle to find a way into the clinical workflow. Part of the reason for the lacking translation into the clinical world could be an error of analyzing images that do not contain sufficient information. Errors in positioning leading to insufficient image quality are common, and correct positioning is crucial for assessment [25]. The sensitivity of the assessment of mammograms drops by 21% among cases with failed positioning compared to cases with correct positioning [11]. Audits performed in the USA showed insufficient positioning as the leading cause for failed unit accreditations in ACR-accredited faculties, being responsible for 79% of all errors [26].

Current studies of quality control in breast cancer diagnosis focus on quality assurance. CNNs potentially allow for automated image quality assessment according to European Reference Organization for Quality Assured Breast Cancer Screening and Diagnostic Services (EUREF) [27]. However, the number of studies focusing on specific features visible on images, impact image quality is rather limited. Subjectivity of visual assessment is limiting the consensus within radiographers and radiologists while assessing image quality [8]. Lack of full harmonization and low adherence to ACR and EU guidelines result in a wide variability of results and openness to interpretation [28]. Lack of evidence-based practice and insufficient equipment for assessment lead to a situation where 44% of radiographers are not fully aware of the guidelines to be followed in their practice [29]. This lack of standardization is visible when analyzing tomosynthesis images. Due to lack of widely accepted guidelines for modern imaging techniques, synthetic 2D reconstructions from tomosynthesis were used for evaluation and visualization. Initial studies have been launched to apply AI to other techniques showing promising preliminary results in automated breast ultrasound [30] and breast CT [31]. AI solutions show promise of a significant reduction of workload for technicians while allowing standardization of procedures [32]. Image quality assessment could also be beneficially applied to diagnostic mammographies; however, in symptomatic patients with, e.g. mastodynia, signs of inflammation or post-operative scars it might prove difficult to apply the same standards as used in a screening setting. Therefore, dedicated guidelines for diagnostic mammographies might be necessary.

Our study has some limitations: (1) Datasets chosen for training, validation and testing may potentially be biased due to the retrospective nature of this study. (2) Images in this study came from a limited number of manufacturers, which may have an effect on a number of possible variants of images used for analysis. However, the purpose of this study was to provide a proof-of-principle of automated quality assessment and to obtain initial experiences on achievable accuracies. (3) Not all ethnicities were included in this study; however, representative data were taken from the general cohort of female patients in our institutions. (4) In this study, no other architectures of AI algorithms have been evaluated. Therefore, it might be possible to achieve even higher accuracies with other dCNN configurations. (5) This is a retrospective study focusing on evaluation of single-quality features. To evaluate an impact of real-time feedback on the performance of diagnostic units, an additional prospective study shall be conducted.

In conclusion, this study has proven the feasibility of automated assessment of quality features with the trained AI models that allow for standardization of quality control. The clinical implementation of our solution may allow for fast, observer-independent feedback to radiographers and radiologists in the assessment of the overall image quality for breast positioning. This may improve the sensitivity and specificity of examinations and reduce errors, recalls and workload for radiologists and technicians.

## Data Availability

Not applicable.

## Abbreviations

ACR: American College of Radiology
AI: Artificial intelligence
CC: Cranio-caudal
dCNN: Deep convolutional neural network
DM: Digital mammography
IMF: Inframammary fold
MLO: Medio-lateral oblique
PGMI: Perfect–Good–Moderate–Inadequate quality assessment scoring system
QA: Quality assurance
QC: Quality control
RMSE: Root mean squared error
